# The Role of Mitophagy in Skeletal Muscle Damage and Regeneration

**DOI:** 10.3390/cells12050716

**Published:** 2023-02-24

**Authors:** Eirini Chatzinikita, Maria Maridaki, Konstantinos Palikaras, Michael Koutsilieris, Anastassios Philippou

**Affiliations:** 1 Department of Physiology, Medical School, National and Kapodistrian University of Athens, 115 27 Athens, Greece; 2 Faculty of Physical Education and Sport Science, National and Kapodistrian University of Athens, 172 37 Athens, Greece

**Keywords:** exercise, mitophagy, mitochondrial biogenesis, mitochondrial network, myogenesis, muscle damage, regeneration

## Abstract

Mitochondria are cellular organelles that play an essential role in generating the chemical energy needed for the biochemical reactions in cells. Mitochondrial biogenesis, i.e., de novo mitochondria formation, results in enhanced cellular respiration, metabolic processes, and ATP generation, while autophagic clearance of mitochondria (mitophagy) is required to remove damaged or useless mitochondria. The balance between the opposing processes of mitochondrial biogenesis and mitophagy is highly regulated and crucial for the maintenance of the number and function of mitochondria as well as for the cellular homeostasis and adaptations to metabolic demands and extracellular stimuli. In skeletal muscle, mitochondria are essential for maintaining energy homeostasis, and the mitochondrial network exhibits complex behaviors and undergoes dynamic remodeling in response to various conditions and pathologies characterized by changes in muscle cell structure and metabolism, such as exercise, muscle damage, and myopathies. In particular, the involvement of mitochondrial remodeling in mediating skeletal muscle regeneration following damage has received increased attention, as modifications in mitophagy-related signals arise from exercise, while variations in mitochondrial restructuring pathways can lead to partial regeneration and impaired muscle function. Muscle regeneration (through myogenesis) following exercise-induced damage is characterized by a highly regulated, rapid turnover of poor-functioning mitochondria, permitting the synthesis of better-functioning mitochondria to occur. Nevertheless, essential aspects of mitochondrial remodeling during muscle regeneration remain poorly understood and warrant further characterization. In this review, we focus on the critical role of mitophagy for proper muscle cell regeneration following damage, highlighting the molecular mechanisms of the mitophagy-associated mitochondrial dynamics and network reformation.

## 1. Introduction

Mitochondria are cell organelles that possess a critical role in cellular metabolism and function, generating most of the cell’s energy, while they also control cell growth and apoptosis. Damage to these structures makes them defective and has been associated with a wide variety of diseases [1]. Since damaged mitochondria can disrupt cellular homeostasis, quality control systems have been developed to clear the damaged mitochondria, through selective autophagy and degradation (mitophagy), and to protect cell energy metabolism [2]. Mitophagy prevents the buildup of injured mitochondria, which would otherwise cause cellular disruption by reactive oxygen species (ROS)-induced oxidation and/or cell death [3,4]. Mitophagy, fission/fusion, mitochondrial biogenesis, cellular energy metabolism, and cellular stress sensors cooperate to sustain mitochondrial function [5,6,7].

**Figure 1:**
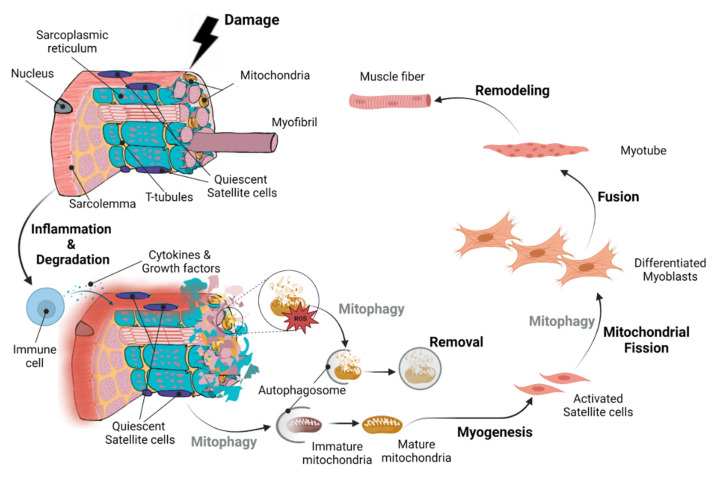
**Overall sequence of events during skeletal muscle regeneration and remodeling following damage.** Extensive intracellular mitochondrial modification (i.e., mitochondrial turnover and clearance as well as mitochondrial network reformation) is required in parallel with satellite cell activation and differentiation to repair or generate new myofibers. Mitophagy plays a key role in the repair and/or formation of new muscle fibers through muscle regeneration and myogenesis. Under normal conditions, satellite cells, which are skeletal muscle precursor cells, remain in a dormant state (quiescent satellite cells) between the basal lamina and sarcolemma of muscle fibers. After muscle damage, these cells become active through the influence of cytokines and growth factors released by immune cells. Quiescent satellite cells go through a process of mitochondrial network remodeling (from immature to mature mitochondria) via mitophagy to create activated satellite cells, also known as myoblasts. As myoblasts differentiate and mature, they require further metabolic remodeling through mitochondrial fission and mitophagy. The differentiated myoblasts can then fuse directly with the damaged myofibers to restore their structure and function, or fuse with each other to form myotubes that eventually develop into skeletal muscle fibers.

A complex network of tube-like structures characterizes the morphological and functional properties of mitochondria, and this network undergoes a dynamic remodeling in skeletal muscle in response to various conditions, such as exercise and muscle damage. Mitophagy can regulate skeletal muscle growth and regeneration processes through the regulation of the mitochondrial network [8]. Interestingly, quiescent muscle satellite cells, which sustain regeneration of skeletal muscle following damage, have also been found to exhibit mitophagic activity as they activate, proliferate, and differentiate to repair damaged muscle cells [9] (Figure 1). In this review, we focus on the critical role of mitophagy for proper muscle cell regeneration and function following damage, highlighting the molecular mechanisms of mitophagy-associated mitochondrial dynamics and mitochondrial network reformation.

## 2. Autophagy

Autophagy, a term coming from the Greek words “auto” and “phagy” meaning self-eating, is a self-degradative process of the cell that removes any damaged, dysfunctional or unnecessary components through a lysosome-dependent regulated mechanism. Macro-autophagy, a type of autophagy, is the cellular process that refers to the formation of autophagosomes, which are double-membrane vesicles that enclose parts of organelles, cytoplasm, and/or protein aggregates before their transportation to lysosomes for destruction. This catabolic process is critical for cell survival and contributes to various biological functions [10,11]. Under normal conditions, autophagy mainly inhibits the accumulation of damaged organelles and unfolded proteins, while it is characterized as a pro-survival mechanism in response to physiological stress by providing metabolic substrates [12,13]. From the formation of autophagosomes to the degradation of their components (macro-autophagy), there is a succession of different stages with high energy requirements, and more than 30 autophagy-related proteins (ATGs) are involved in these sequential phases [14]. Briefly, the activation of Unc-51-like autophagy activating kinase 1 (ULK1), as well as the production of phosphatidylinositol 3-phosphate (PI3P), is required for the initiation and expansion of the phagophore, the double-membrane structure that ultimately generates a completed autophagosome. Furthermore, vesicles containing ATG9 supply extra lipid-membrane substrates for pre-autophagosomal formation [11,15,16]. Then, autophagosome elongation and maturation require the ATG12 coupling system, which comprises the ATG5, ATG7, ATG10, ATG12, and ATG16L1 proteins [17,18,19]. ATG12 and ATG5 are covalently coupled by two enzymes, ATG7 and ATG10 [17,20,21]. The ATG12–ATG5 protein complex then generates a complex with ATG16L1 in the pre-autophagosomal structure, and the E3-like activity of the ATG12–ATG5–ATG16L complex enhances the interaction of Microtubule-associated protein light chain 3 (LC3) with phosphatidylethanolamine (PE) to create LC3-PE (LC3-II). When the autophagosome unites with the lysosome, the LC3-II protein is rapidly degraded [22,23].

Autophagosomes have been detected practically in every type of muscle dystrophy and myopathy examined so far, and they are a hallmark of a group of muscle diseases known as autophagic vacuolar myopathies [24]. An increase in polyubiquitinated proteins has been found in autophagy-null muscles in tissue-specific autophagy knockout animals [25,26,27,28]. These findings, along with evidence that proteasome activity is not significantly impaired in ATG7-deficient muscles, implies that some ubiquitinated proteins are selectively targeted for lysosomal destruction by autophagy [29,30,31]. Moreover, selective inhibition of autophagy in skeletal muscle tissue, through ATG7 knockout, was found to cause atrophy, muscle weakness, and other symptoms of myopathy [32]. Another animal model of autophagy inhibition, the muscle-specific ATG5 knockout mouse, revealed a similar atrophic phenotype [33]. Conversely, transgenic ATG5 overexpression has been demonstrated to improve functional ability and lengthen longevity in mice, most likely due to increased autophagy [34].

## 3. Mitophagy

**Figure 2:**
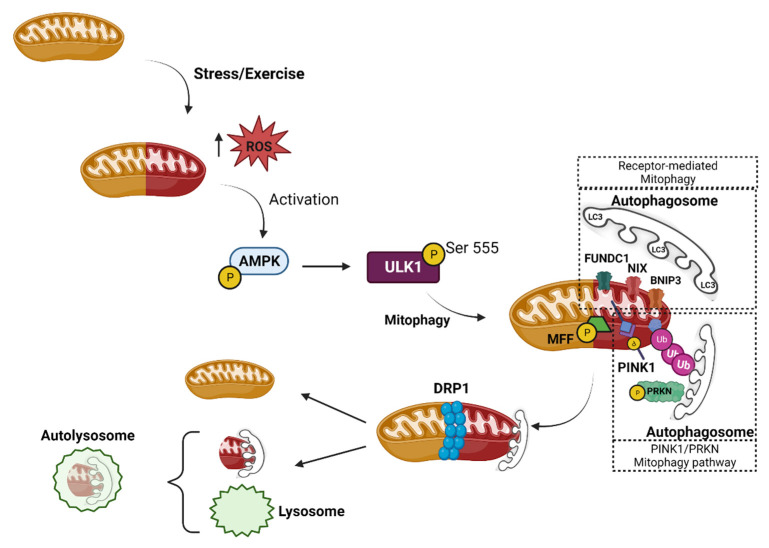
**Exercise-induced mitophagy.** During exercise-induced energetic/oxidative stress, the damaged and dysfunctional mitochondria (represented by the transition of their color from orange to red) are removed from muscle cells. Acute exercise causes mitochondrial stress and activates the removal of damaged/dysfunctional reticulum regions via mitochondrial fission and mitophagy. When the inner mitochondrial membrane potential is disrupted, the production of mitochondrial-derived reactive oxygen species (mtROS) increases, activating the AMP-activated protein kinase (AMPK)/Unc-51-like autophagy activating kinase 1 (ULK1) signaling pathway. While PTEN-induced kinase 1 (PINK1) phosphorylates Parkin (PRKN) to initiate the mitophagy process (PINK1/PRKN pathway), the exercise-induced AMPK activation promotes the formation of autophagosomes through an ULK1-mediated mechanism. AMPK phosphorylates mitochondrial fission factor (MFF) to stimulate mitochondrial fission through the activation of dynamin-related protein 1 (DRP1). The autophagosome/mitochondria complexes then fuse with lysosomes to complete the degradation of the damaged mitochondria.

Mitophagy is a type of macro-autophagy in which dysfunctional mitochondria are selectively removed through the autophagy–lysosome complex. Broken and depolarized mitochondria are eliminated from healthy skeletal muscle cells via the mitophagy pathway [35]. To assist in minimizing cellular damage and preserve homeostasis, many quality control systems, including autophagic clearance of damaged mitochondria, have been identified. Mitophagy exists at the crossroads of cell survival and death [3,36], while mitophagy failure has been linked to a variety of pathological conditions and disorders [37,38]. Mitochondria may undergo mitophagy via a variety of mechanisms, and these combinative mechanisms highlight the evolutionary significance of removing damaged mitochondria. Mitophagy is started by the same mechanism as the autophagy initiation pathway. Specifically, the ULK1 complex is colocalized with ATG9, and both are required for mitophagy initiation [39]. The ULK1 complex transmits the stress-induced AMP-activated protein kinase (AMPK) signals to foster autophagy induction, controlled via phosphorylation (Figure 2), while the ATG9 vesicles provide another lipid membrane that will become part of the de novo formed autophagosome [40,41].

More specifically, basal mitophagy is a continuous process that guarantees the recycling of the fragmented, damaged mitochondria and occurs in most cell types during regular mitochondrial maintenance. Its extent varies depending on cell and tissue type, with cardiac and skeletal muscle exhibiting high levels of basal mitophagy [42]. These muscle cells have evolved well-coordinated quality control systems, including mitochondrial synthesis, mitochondrial dynamics, and mitophagy [43,44,45,46,47]. In particular, cardiac mitochondria produce massive quantities of adenosine triphosphate (ATP) via oxidative phosphorylation (OXPHOS) to sustain contractile activity, although this generates a lot of oxidative stress [48]. Stress signals influence mitochondrial function and cause acute mitochondrial clearance due to the stress-induced mitophagy [49] (Figure 2). Specifically, mitophagy is strongly activated under stress and contributes to mitochondrial quality control to mediate stress-induced metabolic adaptations. Mitophagy removes degraded mitochondria and new organelles are generated via mitochondrial biogenesis, which are specifically designed to function under physiological stress. Pathways responsible for the degradation of mitochondria and those responsible for mitochondria regeneration can function simultaneously (Figure 1).

**Figure 3:**
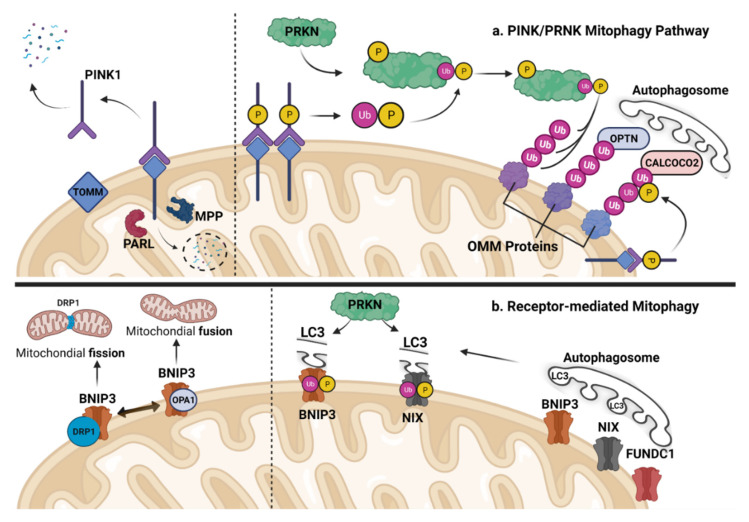
(**a**) **The PINK1/PRKN mitophagy pathway.** Under normal conditions, PTEN-induced kinase 1 (PINK1) partially enters mitochondrion, where it is severed by presenilin-associated rhomboid-like protein (PARL) and mitochondrial processing peptidases (MPPs), and then released into the cytosol to be degraded (left side of dashed line). However, when the inner mitochondrial membrane potential collapses (right side of dashed line), PINK1 can build up on the outer mitochondrial membrane, triggering parkin RBR E3 Ubiquitin Protein Ligase (PRKN) phosphorylation both directly and indirectly through ubiquitination. The highly activated PRKN can then mark outer mitochondrial membrane (OMM) proteins with ubiquitin, making them recognizable to cargo proteins such as optineurin (OPTN) and coiled-coil domain 2 (CALCOCO2), which leads to their degradation through the process of autophagy. (**b**) **Receptor-mediated mitophagy.** Specific receptors target damaged mitochondria for removal. BCL2 interacting protein 3 (BNIP3), NIX (BCL2 Interacting Protein 3-Like), and FUN14 domain containing 1 (FUNDC1) are examples of these receptors that are located on the outer mitochondrial membrane and interact directly with microtubule-associated protein light chain 3 (LC3), a protein involved in autophagy, to eliminate damaged mitochondria. The interaction between NIX and BNIP3 with LC3 is enhanced by their phosphorylation. While FUNDC1 does not interact with PINK1/PRKN to cause mitophagy, the ubiquitination of NIX and BNIP3 receptors by PRKN highlights the complex relationship between receptor-mediated mitophagy and the PINK1/PRKN mitophagy pathway. These receptors promote the division of damaged organelles by breaking down and releasing optic atrophy gene 1 (OPA1) and by attracting to the mitochondrial surface the dynamin-related protein 1 (DRP1), a protein that mediates mitochondrial fission through the scission of the outer mitochondrial membrane (left side of dashed line). OPA1 can activate DRP1, which then induces the division of mitochondria. This division is essential for the removal of damaged mitochondria and the maintenance of mitochondrial quality control. On the other hand, OPA1 is also involved in the fusion of the inner mitochondrial membrane and is required for the maintenance of the normal cristae structure and respiratory function of mitochondria. The fusion of mitochondria by OPA1 is inhibited by the actions of DRP1, ensuring that the division and merging processes are properly balanced.

In skeletal muscle, mitophagy can be mediated by two different mechanisms: (1) the Parkin (PRKN) and PTEN-induced kinase 1 (PINK1) pathway (Figure 3a); and (2) receptor-mediated mitophagy (Figure 3b). Other mechanisms of mitochondrial quality control have also been proposed [50], such as the mitochondrial proteins’ selective degradation by the ubiquitin proteasomal system (UPS) [51,52] or transfer of the tiny mitochondrial-derived vesicles (MDVs) to the lysosome [53]; however, the role of these additional mechanisms of mitochondrial quality control remains to be characterized in skeletal muscle.

The PINK1/PRKN pathway is the most characterized mitophagy mechanism; PINK1 is a Ser/Thr kinase that acts as a molecular detector for depolarized or damaged mitochondria [54]. The translocase of the outer mitochondrial membrane (TOMM) complex of proteins is located in the cytosol, where full-length PINK1 is often present [55]. In a healthy state, high mitochondrial membrane potential (ΔΨm) permits the positively charged PINK1 with its N-terminal mitochondrial-targeting sequence (MTS) to partially enter the mitochondrial matrix where it is cleaved by mitochondrial processing peptidases (MPPs). After this first PINK1 cleavage, presenilin-associated rhomboid-like (PARL) on the inner mitochondrial membrane (IMM) divides the transmembrane domain, leading to the separation of PINK1 from the TOMM complex [56,57]. (Figure 3a). In low-ΔΨm, the positively charged MTS avoids MPP- and PARL-mediated cleavage of defective mitochondria because it is unable to enter the mitochondrial matrix. Consequently, PINK1 persists in complex with TOMM and eventually builds up on the outer mitochondrial membrane (OMM), where it can be phosphorylated at Ser228 and Ser402 [58], facilitating PINK1-mediated mitochondrial recruitment of PRKN [59]. Once attracted to the OMM, PRKN is phosphorylated at Ser65 and partly activated by PINK1 [55,58,59,60]. With the presence of ubiquitin, which is also phosphorylated by PINK1, PRKN is completely activated [61,62,63]. Numerous target proteins found on the OMM can be ubiquitinated by the fully active PRKN [64,65]. The OMM protein’s supplement of the ubiquitin chain enables the autophagic load-recognition protein optineurin (OPTN), TAX1 binding protein 1 (TAX1BP1), calcium-binding and coiled-coil domain 2 (CALCOCO2), sequestosome 1 (SQSTM1) and neighbor of BRCA1 gene 1 (NBR1) to recognize damaged mitochondria [51,66] (Figure 3a). These recognition proteins can subsequently interact with the autophagosome’s lipidated light chain 3 (LC3-II) or gamma-aminobutyric acid receptor-associated protein (GABARAP), which are ATG8 homologs, causing immersion and movement to the lysosome for destruction.

The receptor-mediated mitophagy via receptors located on the OMM is another mitophagy mechanism, which has been shown to be activated by starvation or hypoxia [67,68]. Hypoxia-induced mitophagy affects transcriptional and post-translational regulation of mitophagic receptors [68,69,70]. Specifically, several hypoxia-inducible proteins, which were first demonstrated to be significant in apoptosis [71], such as FUN14 domain containing 1 (FUNDC1), B-cell lymphoma 2 (BCL2) interacting protein 3 (BNIP3), and BCL2 interacting Protein 3-Like (BNIP3L/NIX), have also been found to be crucial in receptor-mediated mitophagy [72,73,74,75,76] (Figure 3b).

More specifically, under hypoxia, BNIP3 and BNIP3L/NIX are located at the OMM in response to depolarization stimuli and have been linked to apoptosis [77]. The buildup of these proteins on the OMM causes mitochondrial outer membrane permeability (MOMP), due to interaction with pro-apoptotic proteins, to begin additional depolarization of the mitochondria [78,79,80], thus activating the intrinsic apoptotic cascade [79,81]. These molecules have also been found to increase autophagy via the replacement of BCL2 and BCL2-like 1 (BCL2L1) protein by Beclin-1 (BECN1), causing the creation of the autophagosomal complex, while BNIP3 and BNIP3L/NIX removal greatly inhibits hypoxia-induced autophagy [51]. Although a receptor-mediated mitophagy program is thought to be primarily responsible for hypoxia-induced mitochondrial clearance [68,76,82], a possible interaction with PINK1/PRKN pathway has been also proposed [68,76,82,83] (Figure 3b). Thus, BNIP3 has been reported to interact with mitochondrial-localized PINK1 and block its deterioration, which enhances PINK1/PRKN-mediated mitophagy [83]. BNIP3 attaches to and limits the cleavage of bound PINK1–OMM, hence boosting full-length PINK1 buildup and mitophagy through the PINK1/PRKN pathway. On the other hand, BNIP3L appears to be a key component in mitochondrial clearance in the absence of PINK1/PRKN [80].

Another OMM protein that is synthesized in a hypoxic environment is FUNDC1, a phosphorylation-regulated protein that can act independently of PINK1/PRKN. Specifically, phosphorylation of FUNDC1 at Ser13 and Tyr18 reduces its activity [74,84], while its phosphorylation at Ser17 by ULK1 increases FUNDC1 interaction [75]. Interestingly, although FUNDC1 does not interact with PINK1/PRKN to cause mitophagy when under stress (Figure 3b), nevertheless in response to hypoxia, dephosphorylation of FUNDC1 at Ser13 by phosphoglycerate mutase 5 (PGAM5) mediates mitophagy [84]. On the other hand, FUNDC1 ubiquitination results in its degradation and thus mitophagy reduction [85]. Moreover, the interaction of FUNDC1 with calnexin controls mitophagy and mitochondrial fission, whereas its absence circumvents mitophagic degradation via hyperfusion [86]. Overall, the balance between the phosphorylation and ubiquitination states of FUNDC1, as well as the regulation of mitochondrial fission, controls FUNDC1-mediated mitophagy.

The aforementioned mitophagy-mediating receptor proteins are primarily regulated by the transcription factors hypoxia-inducible factor-1α (HIF-1α) and forkhead box O3 (FOXO3) [76,87,88,89]. The degree of receptor-mediated mitophagy may be determined by the interaction of transcriptional and translational regulators of mitophagy-mediating receptors. Indeed, direct transcriptional activation of BNIP3L was found to be induced by protein kinase C alpha (PRKCA) signaling of trans-acting transcription factor SP1 [90]. In addition, the mitophagy-mediating receptors residing on the OMM interact directly with the autophagosome’s ATG8 homologs, such as GABARAP and LC3-II, to drive mitophagy, although these mitophagy receptors exhibit different affinities for ATG8 homologs [72,73,74,75,76].

## 4. Muscle Damage and Mitophagy

Muscle damage affects muscle homeostasis, inducing disruption of the myofibril structure, reduced sarcolemma stability, and segmental necrosis of myofibers [91]. In general, tissue damage in a sterile environment does not necessitate the activation of an acquired immune response and is divided into two phases: the pro-inflammatory phase, with leukocytes penetrating the injured area; and the anti-inflammatory phase, in which the tissue regenerates or scars depending on the qualities of its parenchymal cells. Unique aspects of the inflammatory response have been characterized, which depend on the tissue type and the kind of damage (e.g., traumatic, toxic, or ischemic injury) [92].

Muscle damage caused by strains, contusions, lacerations, ischemia, burns, or even severe exercise can disturb critical cellular processes [93]. Intramuscular inflammatory signaling is important in modulating the regeneration response to muscle fiber damage. [94,95]. A brief inflammatory response initiates a pro-myogenic signaling cascade that contributes to muscle repair, remodeling, and maintenance [93,96,97,98] (Figure 1). Tissue healing requires the resolution of inflammation, i.e., the transition from the inflammatory to the anti-inflammatory phase. This process is primarily governed by macrophages, which are the primary orchestrators of the inflammatory response following damage [99]. In this phase, energy metabolism and mitophagy are expected to play critical roles in macrophage homeostasis and inflammasome induction [100].

Neutrophil invasion of the damaged muscle tissue is the primary driver of the anti-inflammatory phase, which starts immediately after the injury. These cells eliminate waste by phagocytosis, and they also exude chemotactic chemicals to attract macrophages, which phagocytose and clear muscle tissue debris [101,102]. Myogenesis, the second phase of the regeneration process, depends on the initial presence of the phagocytic, pro-inflammatory M1 macrophages and the effective shift to the non-phagocytic, anti-inflammatory M2 macrophages [102,103,104]. Satellite cells, the quiescent skeletal muscle stem cells, receive signals from macrophages that activate them and they initially proliferate before undergoing myogenic differentiation. Differentiated satellite cells (myoblasts) either fuse to the damaged muscle fibers to complete their repair, or proliferate, differentiate and fuse with myotubes to create new muscle fibers [104,105,106,107] (Figure 1). Depending on the severity/type of damage and the state of the cells involved, complete skeletal muscle regeneration may last from 14 to 28 days or longer [97,98,108,109,110].

Interestingly, extensive intracellular modification of the mitochondria is required during the satellite cells’ activation and differentiation to repair or generate new myofibers (Figure 1), and the ability of myoblasts to differentiate and of skeletal muscle tissue to regenerate is diminished in the absence of mitochondrial remodeling [79,111,112,113,114,115,116,117].

Many metabolic byproducts, such as reactive oxygen species (ROS), are frequently recognized as the primary cause of cellular damage. ROS are responsible for generating mitochondrial dysfunction and vice versa, while oxidative stress-induced damaged mitochondria must be removed through mitophagy [109,118] (Figure 2). Evidence suggests that increased levels of mitochondria-derived ROS (mtROS) production and mtDNA release cause inflammation, which accelerates mitochondrial failure. Specifically, the principal ROS reservoirs are damaged and result in dysfunctional mitochondria, which produce mtROS during the oxidative phosphorylation process. ROS cross the OMM and activate redox-sensitive transcription factors, hence increasing the production of pro-inflammatory proteins [110]. Moreover, it has been observed that mtROS cause the mitogen-activated protein kinase (MAPK) activation to be maintained by blocking MAPK phosphatases [119]. In addition, mtROS are linked to inflammation via the NLR family pyrin domain containing 3 (NLRP3) inflammasome. Interestingly, it has been shown that inhibition of mitophagy results in the buildup of mtROS and damaged mitochondria, which enhances the NLRP3 inflammasome activation [120]. Moreover, voltage-dependent anion-selective channel 1 (VDAC1) on the OMM is needed for mtROS generation and, as a key ion channel, it influences the assembly and activation of the NLRP3 inflammasome by regulating Ca^2+^ flow [120,121]. In addition, the classical autophagy receptor, p62, is upregulated in NLRP3 inflammasome-activated cells and is translocated to the damaged mitochondria to stimulate mitochondrial priming and enhance mitophagy [122,123]. Overall, mitophagy is not only an important process for removing excess mtROS and defective mitochondria but is also an inflammatory factor that may control mitophagy. Moreover, it appears that mitophagy has self-limiting systems for regulating the pro-inflammatory response and minimizing oxidative cellular damage [109].

## 5. Muscle Regeneration and Mitophagy

The complex regeneration process following muscle damage is controlled by the interaction of several cellular and molecular processes [124,125,126], and autophagy has been shown to be a crucial component needed for skeletal muscle regeneration. A delayed regeneration response to myotoxin-induced muscle damage has been shown to be caused by defective autophagy [114]. AMP-activated protein kinase (AMPK), BECN1 and unc-51-like kinase 1 (ULK1) were revealed to be indicators of enhanced autophagy following skeletal muscle damage [114]. Additionally, higher BNIP3 expression was observed in skeletal muscle during its regeneration, along with higher levels of PRKN, PINK1 and autophagosome translocation in the damaged muscle’s mitochondria seven days after freezing injury [127]. Moreover, muscle regeneration was found to be significantly delayed, along with inflammation and fibrosis, in PRKN-deficient rats after myotoxic damage [128], indicating that defective satellite cells, muscle fibers, immune or fibrogenic cells, all of which contribute to the regenerative response, may be partially responsible for the poor muscle regeneration following damage in the PRKN-deficient animals [126,129,130].

Recent research has revealed that mitophagy is a mechanism that controls cell fate via metabolic reprogramming [131]. Selected mitochondrial populations in progenitor cells and stem cells can be removed to induce phenotypic changes in these cells, such as differentiation [131] or dedifferentiation [132]. In other words, the dynamic remodeling of the mitochondrial network and mitophagy alter the bioenergetic profile of cells, and programmed mitophagy particularly modifies glycolysis, beta-oxidation, and OXPHOS [131]. The quantities of metabolic intermediates needed for the transcriptional control of cell destiny are also changed in conjunction with this shift in the bioenergetic profile [133,134,135], which is most noticeable during myogenesis [101]. Overall, mitophagy is crucial at the beginning of myogenesis, controlling cell destiny, while adult muscle regeneration and the function of muscle stem cells are also governed by mitophagy (Figure 1).

### 5.1. Muscle Regeneration and Mitochondrial Biogenesis

Mitochondrial biogenesis is a tightly controlled process that depends on the synchronized interplay between mitochondrial and nuclear molecules. Specifically, peroxisome proliferator-activated receptor (PPAR)-gamma coactivator-1 (PPARGC1) has been characterized as a key regulator of mitochondrial metabolism and biogenesis [136], while PPARGC1A, PPARGC1B, and PPARG-related coactivator 1 (PPRC1) also belong to the PPARGC1 family [122]. These factors, along with nuclear receptors and transcriptional regulators, comprise the cell metabolic machinery [137]. In particular, PPARGC1A, also known as PGC1, has been assumed to be a required mediator of biogenesis [138], although PPARGC1B and some metabolites are emerging factors that have been investigated for their potential role in the PPARGC1A-independent mitochondrial biogenesis [139,140,141,142].

The co-transcriptional regulatory factor PPARGC1A is strongly inducible by a variety of physiological circumstances, such as cold, fasting, hypoxia, and exercise [143]. These situations affect energy levels and/or stress signaling cascades, eventually causing mitochondrial biogenesis. In these settings, numerous interrelated pathways are triggered to satisfy the demands of muscle cells/tissues and promote survival, resulting in elevated levels of PPARGC1A [144,145]. PPARGC1A can also induce adaptations by promoting the expression of metabolism and mitochondrial biogenesis genes [144,145]. PPARGC1A canonical transduction results in its interaction with nuclear respiratory factors, which are important regulators of mitochondrial biogenesis. These factors have been shown to be important in targeting a wide range of genes involved in detoxification, mitochondrial membrane transport, OXPHOS, and mitochondrial DNA replication [146].

On the other hand, PPARGC1B has been assumed to be an essential regulator of mitochondrial biogenesis in skeletal muscle since it exhibits comparable features to PPARGC1A [147,148]. Specifically, PPARGC1B, like PPARGC1A, can interact with nuclear respiratory factors to increase the expression of mitochondrial transcription factors and initiate mitochondrial biogenesis in skeletal muscle cells [139,141]. Interestingly, skeletal muscle mitochondrial function is significantly reduced in double PPARGC1A- and PPARGC1B- knockout mice compared with the elimination of either alone [149].

### 5.2. Mitophagy and Mitochondrial Dynamics in Skeletal Muscle Maintenance

Mitochondria are extremely active cell organelles that alternate their status between fission (splitting) and fusion (merging). Fission is the process by which a single mitochondrion divides into two or more smaller mitochondria. Fission allows the removal of damaged or unnecessary mitochondria through mitophagy and the distribution of mitochondria to different regions of the cell. This process is mediated by dynamin-related protein 1 (DRP1), which is recruited to the mitochondrial outer membrane by the mitochondrial fission protein 1 (FIS1) [97]. DRP1 then forms a ring-like structure around the mitochondrion, which constricts and divides the organelle into smaller fragments [108,150] (Figure 2 and Figure 3). On the other hand, fusion is the process by which two or more mitochondria merge to form a single, larger mitochondrion. Fusion is responsible for the mixing of contents between mitochondria, promoting the distribution of proteins and lipids, and increasing the efficiency of energy production. This process is mediated by Mitofusin 1 and 2 (MFN 1 and 2), which interact and bring the outer membranes of the mitochondria into proximity [111]. The inner membrane then fuses, creating a single, interconnected network of mitochondria [151,152].

Fission and fusion are essential for mitochondrial maintenance (of shape and function) and so are important for cellular viability [146]. To ensure that mitochondria are distributed evenly in each daughter cell, mitochondrial fission is essential during cell division [153]. Fission also allows for the effective clearance of damaged mitochondria through selective autophagy (mitophagy) or by contributing to cell death induction [154,155]. Proteins that target the outer membrane, such as FIS1, mitochondrial fission factor (MFF), and mitochondrial dynamic proteins 49 and 51 (MiD49 and MiD51), contribute to mitochondrial fragmentation [156]; nevertheless, DRP1 is largely responsible for mitochondrial fission [151,152] (Figure 2 and Figure 3).

Conversely, mitochondrial fusion is required for mtDNA inheritance and aids in stress mitigation by combining contents from damaged/dysfunctional and healthy mitochondria in a commensurate manner [157,158]. Mitochondrial fusion is also necessary during cell differentiation and inhibits mitochondrial autophagy (mitophagy) [159], while the OPA1 protein and MFN-1 and MFN-2 primarily govern mitochondrial fusion [151,152] (Figure 3b). Together, fission and fusion play an important role in the maintenance of the mitochondrial network and its function, allowing cells to adapt to changes in energy demands, the balance between damaged and functional mitochondria, and in the distribution of these organelles to different regions of the cell. Over the recent years, several studies have shown the role of mitochondrial dynamics in the maintenance of skeletal muscle and mitochondrial structure and function. Specifically, mice missing both MFN-1 and MFN-2 in skeletal muscle exhibit significant mitochondrial malfunction and buildup of mitochondrial DNA damage [111]. MFN-2 loss in skeletal muscle has been reported to cause oxidative stress and muscular atrophy in mice [111]. MFN-1 and MFN-2 elimination in adult muscle tissue led to a significant impairment in exercise performance in mice [160]. Moreover, OPA1 deletion causes mitochondrial failure, endoplasmic reticulum stress, inflammation, and oxidative stress in skeletal muscle [116,161,162]. OPA1 deficiency also significantly increases the release of fibroblast growth factor 21 (FGF21) from skeletal muscle fibers, resulting in tissue senescence and inflammation [116]. In addition, overexpression of muscle-specific DRP1 has been demonstrated to impede skeletal muscle development in mice [108,150]. Inhibiting skeletal muscle mitochondrial fission via genetic DRP1 and FIS1 silencing has been demonstrated to prevent muscle wasting caused by starvation or overexpression of the atrophy transcription factor FoxO3a [97]. However, muscle-specific DRP1 deletion results in a severe myopathic phenotype characterized by muscular weakness, wasting, and indications of muscle tissue necrosis and repair [112]. DRP1 deletion and knockdown both modify mitophagy and autophagy [112,129].

Through biogenesis, fission/fusion and mitophagy, cellular energy/nutrient and stress sensors cooperate to sustain mitochondrial function. A significant cell energy sensor is the AMPK, which is activated in low-energy situations [73,163], and its activation is critical in tissues with high energy needs, such as skeletal muscle. AMPK acts through the phosphorylation of multiple energy metabolism targets to maintain cell energy levels [74,80,164]. Specifically, AMPK-mediated phosphorylation of PPARGC1A allows it to interact with co-transcription factors to upregulate mitochondrial biogenesis genes. AMPK has also been demonstrated to phosphorylate different epigenetic regulators, promoting PPARGC1A expression [51], and it also phosphorylates autophagic target proteins. AMPK can directly inactivate the sensor mTOR complex 1 (MTORC1) by phosphorylating the regulatory-associated protein of mTOR, promoting mitochondrial dynamics in skeletal muscle aging [165]. MTORC1 inactivation and AMPK-mediated phosphorylation of FoxO3 modulates multiple autophagy (ULK1, BECN1) and mitophagy (BNIP3, BNIP3L) factors [166,167,168]. AMPK also phosphorylates ULK1 directly, inducing autophagosome formation in skeletal muscle [169] (Figure 2). Furthermore, AMPK was found to phosphorylate PINK1 to promote PRKN recruitment and mitophagy in aged muscle [170].

### 5.3. Muscle Regeneration, Mitophagy and Mitochondrial Fission

Myoblast differentiation during regeneration and myogenesis is accompanied by metabolic reprogramming, which ultimately leads to an increase in OXPHOS and mitochondrial mass to sustain the developing myotubes. Mitophagy and mitochondrial fission are two components of myogenic differentiation. Myoblasts contain immature mitochondria, which may be characterized by their undeveloped cristaea and low levels of β-oxidation and total respiration [103,105,107,171]. Myoblasts need to produce ATP at a greater rate in response to differentiation-related stimuli to sustain the intracellular remodeling that takes place during differentiation. As a result, differentiated myoblasts change their phenotype to become more oxidative [172,173]. Moreover, myoblasts’ existing mitochondria must be replaced in response to this metabolic alteration. DRP1 levels, autophagy, and mitophagy markers, have all been shown to initially and dramatically rise in response to differentiation stimuli in myoblasts. Moreover, it has been demonstrated that both an increase in mitochondrial fission and mitophagy are necessary for myoblast differentiation [36,174,175,176,177]. Furthermore, chemical inhibition of DRP1 resulted in decreased DRP1 translocation to the mitochondria, lowering fission and, consequently, impairing differentiation and formation of myotubes [174]. Similar results have been reported when ATG7 or BNIP3 were knocked down or deleted in C2C12 myoblasts [175] and greater apoptotic activity was observed as a result of defective fission/mitophagy [35,175]. Myoblasts with ATG7 knockdown or BNIP3 deletion exhibited changed DRP1 and OPA1 levels, implying the involvement of autophagy/mitophagy and mitochondrial dynamics [174]. The significance of mitochondrial fission and subsequent mitophagy in the evolution of myoblast differentiation has been revealed, and studies on skeletal muscle tissue have clearly demonstrated the crucial role of mitochondrial fission for maintaining and increasing muscle mass [97,98,108,112,115]. Reduced mitophagy and severe muscle wasting are caused by skeletal muscle tissue-specific DRP1 knockout [112], although myogenic differentiation 1 (MYOD1) and myogenin (MYOG), two crucial myogenic regulatory factors (MRFs), are unaffected by DRP1 loss in neonatal mice [112]. On the other hand, increased mitophagy caused by overexpression of DRP1 and/or FIS1 resulted in decreased mtDNA content and failure of mitochondrial respiration [97,98]. Another study on skeletal muscle tissue regeneration following cardiotoxin (CTX)-induced muscle damage revealed no changes in the total satellite cell composition or the regeneration program in in vivo myoblast-specific DRP1 knockout mice, indicating that DRP1 does not influence satellite cell function during regeneration [108]. However, FIS1 expression substantially increased between three and five days after a freeze injury of skeletal muscle and remained marginally higher for up to 28 days compared with uninjured/control muscle [178]. Another study showed increased DRP1 levels 14 days after CTX-induced skeletal muscle damage in mice; this was accompanied by higher levels of activated LC3-II, ULK1, and BNIP3, even in mice that received the autophagy inhibitor 3-methyladenine (3-MA) [113]. Moreover, an increase in PINK1/PRKN, BNIP3, and DRP1 coupled with mitochondrial localization of LC3B-II was observed in regenerating muscle seven days after freeze injury [3].

### 5.4. Muscle Regeneration, Mitophagy and Mitochondrial Fusion

Myoblasts differentially express MRFs throughout the differentiation program, with MYOG and MYOD1 being two MRFs whose temporary expression is necessary for the advancement of myoblast differentiation. Increased expression of specific MRFs is accompanied by the elevation of PPARGC1A, mitochondrial transcription factor A (TFAM), COXIV, and mtDNA [3,114,171,175]. Although MYOD1 is produced early in the process of myoblast differentiation, it has been demonstrated that, in the presence of active sirtuin 1 (SIRT1), it can directly increase PPARGC1B transcription or improve PPARGC1A expression [117,179]. Although there is some similarity between the nuclear localization of PPARGC1A and MYOG [177], no definite connection has been shown between them. Poor differentiation is caused by the downregulation of PPARGC1A in C2C12 myoblasts, which also causes increased ROS production, mitochondrial damage, and mitophagy [171]. In fact, through MAPK signaling, ROS generation during myogenic differentiation may be a key factor in boosting mitochondrial biogenesis [172]; however, higher ROS levels appear to have the opposite effect [171]. Increased antioxidant enzyme production during the late differentiation process may significantly lower ROS, which may help reduce mitophagy and allow mitochondrial biogenesis. Additionally, when mitochondrial biogenesis machinery develops and fusion/mitophagy starts to wane, levels of fusion protein OPA1 rise [177]. Notably, the PPARGC1A response is attenuated, and the amount of mitochondrial protein is reduced, when mitophagy is absent in the initial phases of myoblast differentiation [175], highlighting the crucial role of mitophagy for controlling mitochondrial biogenesis. However, enhanced baseline and maximum cellular respiration are required for the highly active myotubes due to mitochondrial synthesis and fusion [81]. Similar to the findings of in vitro studies, MYOG and MYOD1 are increased in skeletal muscle tissue after damage [180,181]. These increases coincide with the overexpression of genes involved in mitochondrial biogenesis, such as protein regulator of cytokinesis 1 (PRC1), PPARGC1B, nuclear factor erythroid 2-related factor 2 (NRF2), nuclear respiratory factor 1 (NRF1), TFAM, and estrogen related receptor alpha (ESRRA) [178,182]. Mitofusin (MFN)1/2 have been shown to play important roles in muscle mass maintenance and the prevention of wasting [112]. MFN1 and MFN2 levels have been shown to rise 3 days after CTX damage [182], whereas MFN2 expression peaks 10 days after freezing injury [178]. Moreover, constitutive skeletal muscle-specific OPA1 deletion in newborn mice was found to cause a large reduction both in quiescent and active satellite cells, though there was no change in apoptosis in these cells, suggesting an association between OPA1 and satellite cell self-renewal [116]. These findings bring out the need for a better understanding of the function of OPA1 in preserving mitochondrial morphology during the various stages of muscle regeneration. Additionally, it has been demonstrated, in freeze- and CTX- induced muscle damage models, that inhibiting mitochondrial protein synthesis by specifically knocking down ESRRA in skeletal muscle considerably hinders regeneration, accompanied by smaller fiber size, decreased mitochondrial density, increased fibrosis, and decreased oxidative enzyme activity in the regenerating skeletal muscle [178,182].

### 5.5. Myogenic Differentiation and Mitophagy

Skeletal muscle fibers have higher bioenergetic requirements than myoblasts; hence, myoblast differentiation and fusion into multinucleated myotubes, and eventually mature myofibers, is accompanied by a significant shift in their metabolic phenotype [103]. Cell destiny is largely determined by the metabolic reprogramming of cells [131]. The resident satellite cells of skeletal muscle are in a quiescent state and they are activated in response to the need to promote regeneration (myogenesis) [103,183,184]. Metabolic reprogramming involves multiple pathways and is an essential aspect of satellite cell activation. Autophagy inhibition, which then inhibits mitophagy, prevents the remodeling of the mitochondrial network and the initiation of mitochondrial biogenesis, processes that are necessary to cover the bioenergetic requirements of myoblasts during myogenic differentiation [171]. Recent research reveals that mitophagy, by enhancing the remodeling of “underdeveloped” mitochondria, plays a significant role during the early stages of myogenic differentiation [45,73,171]. In vitro experiments using C2C12 myoblasts have revealed that mitophagy is often enhanced soon after starting differentiation [171,175,185,186]. With particular regard to the initiation of differentiation in C2C12 cells, several mitophagy pathways have been investigated; however, uncertain findings have been collected about the function of PINK1/PRKN-mediated mitophagy during myoblast differentiation. More specifically, the levels of mitochondrial localized PRKN decreased in response to differentiation stimuli, whereas overall PRKN levels increased during myoblast differentiation [187]. Another study showed that regulating C2C12 myoblast differentiation did not alter the mitochondrial location of PINK1 or PRKN [96]. Moreover, C2C12 myoblasts, investigated under normal, starvation, and heat-shocked conditions, were found not to produce detectable amounts of PRKN, which was exclusively detected in HSP72-knocked-down C2C12 cells [188]. Similarly, PPARGC1A knockdown cells showed higher levels of mitochondrial PINK1 and PRKN during their differentiation [185]. These findings imply that PINK1/PRKN-mediated mitophagy may be reliant on the kind of skeletal muscle cell or the experimental situation, or that it may be more significant in cellular processes other than differentiation, not excluding its potential role in normal C2C12 differentiation. Indeed, during the early stages of differentiation, myoblasts could predominantly rely on receptor-mediated mitophagy to start metabolic reprogramming. Further research on the processes of mitophagy during myogenesis has revealed that remodeling of the mitochondrial network in the early phases of differentiation is regulated by mitophagy [175]. Enhanced vulnerability to cell death, extended oxidative stress, and poor differentiation have been observed in BNIP3-/- myoblasts [175], while low BNIP3 expression is seen in proliferating myoblasts and this expression quickly rises and remains high during myoblast differentiation [175]. Additionally, voltage dependent anion channel 1 (VDAC1), solute carrier family 25 member 4 (SLC25A4), and Cytochrome C, Somatic (CYCS) are present in lower overall levels, along with a PPARGC1A reduction, in BNIP3−/− cells [175]. This is in line with research data showing that bafilomycin A1 therapy inhibits autophagosome fusion, affects autophagy/mitophagy, and consequently lowers PPARGC1A levels during differentiation [171]. Together, these findings imply that mitophagy directly remodels the mitochondrial network by eliminating “immature” mitochondria, most likely through BNIP3, and indirectly promotes a “mature” mitochondrial network by conventional biogenesis pathways [171,175]. Eventually, this reorganization of the mitochondrial network would help myoblasts satisfy their bioenergetic needs during differentiation [171]. Interestingly, a metabolic switch is present in satellite cells during quiescence, proliferation, and differentiation [189]. More specifically, quiescent satellite cells rely heavily on OXPHOS and exhibit a low metabolic rate [190,191], flipping to a higher dependence on glycolysis upon activation or proliferation, and then switching back to mostly OXPHOS with differentiation stimuli [84,86]. The modification of the mitochondrial network by mitophagy may be the cause of this variation in metabolic patterns. Similar to C2C12 myoblasts, quiescent satellite cells require cellular remodeling by autophagy to enable their activation [192].

## 6. Exercise-Induced Muscle Damage and Regeneration

Eccentric exercise, which involves unaccustomed mechanical stress and stretching of skeletal muscle, has been shown to cause muscle injury [193,194]. Exercise-induced muscle damage is associated with transient ultrastructural changes in muscle tissue [195,196], loss of myofiber integrity and leaking of muscle-specific proteins into the blood [96,197,198,199], and reduced muscle function [197,198,199]. Adaptations to muscle-damaging exercise have been related to the common pattern of damage–inflammation–regeneration/remodeling [193,194,200,201], and the dynamics of these physiological processes and their interactions determine the time course and effectiveness of muscle regeneration and remodeling, i.e., whether the inflammation is resolved with scar formation or muscle cell replacement [96,202].

Exercise-induced muscle damage stresses many skeletal muscle organelles, including the mitochondria [124], and mitochondrial damage appears to be a component of the damage caused by eccentric exercise or unaccustomed resistance exercise. However, little is known about the effects of muscle injury on mitophagy and indicators of mitochondrial network remodeling [194,203,204]. The mechanisms of mitochondrial fission, IMM and OMM fusion, and mitophagy are changed in muscle injury-associated mitochondrial damage to restructure the mitochondrial network or eliminate damaged mitochondria [125]. Dysregulated mitochondrial dynamics (fusion and fission) and mitophagy can cause a buildup of damaged mitochondria and oxidative stress [205]. To maintain healthy mitochondria and enhance effective energy generation, the mitochondrial network is continually reorganized through IMM (OPA1) and OMM (MFNs) fusion and fission (DRP1) [127,128], while damaged mitochondria are eliminated by mitophagy after fission (PINK1 and Parkin) [125,130]. In both in vivo and in vitro studies, these processes are crucial for preserving muscle mass [116,129], myofibril contractility [206], muscle force production [116,206], and preventing mitochondrial dysfunction [111,206].

### Exercise-Induced Mitophagy

The possible significance of macro-autophagy in exercise-induced adaptive responses was first recognized after an acute bout of endurance exercise, which was found to result in the conversion of LC3-I to LC3-II and a reduction in p62 levels in skeletal muscle [207]. Similar results were later observed in skeletal muscle mitochondrial fractions with increased BNIP3 expression, providing further concrete proof of the role of macro-autophagy in the removal of mitochondria [208,209]. Similar responses were also shown in mouse myocardium after acute endurance exercise, documenting that exercise-induced mitophagy also occurs in the heart. These results included the enhanced conversion of LC3-I to LC3-II, as well as reduced p62 expression and increased DRP1 expression, shedding some light on the molecular mechanisms through which exercise promotes mitophagy-induced adaptations in striated muscle [207,210].

As research has shown that the resolution of dysfunctional/damaged mitochondria, both in mouse and human skeletal muscle, occurs during the recovery phase (3–6 h) post-exercise rather than during or immediately after acute exercise (0–1 h), mitophagy activation in response to acute exercise requires almost simultaneous activation of many processes, e.g., autophagosome synthesis, mitochondria fission, and autophagosome–lysosome fusion [211,212].

As a “master regulator” of cellular energetics during acute exercise, AMPK has long been investigated and this energy sensor appears to be a crucial factor for the immediate activation of several convergent mechanisms that make up mitophagy [213]. Moreover, ULK1 has been shown to be phosphorylated at Ser555 soon after an acute bout of endurance exercise in mouse skeletal muscle, which is important for the creation of autolysosomes that destroy damaged mitochondria post-exercise [212]. Similarly, two hours of high-intensity cycling exercise in humans resulted in elevated ULK1 dephosphorylation at the inhibitory site Ser757 in the exercised skeletal muscle, demonstrating the activation of the AMPK–ULK1 pathway [214]. It has also been demonstrated that FUNDC1, a possible downstream effector of ULK1, is necessary for mitophagy in murine skeletal muscle, both at rest and in response to exercise-induced energy stress [159,215]. Exercise-induced mitophagy in mouse skeletal muscle depends on the E3 ubiquitin ligase Parkin, which may be attracted by the accumulation of PINK1 [166]. Notably, exercise-induced mitophagy in mouse skeletal muscle did not result in PINK1 stabilization in mitochondria [216]; however, the type of acute exercise used in this study (treadmill running) [212] was mild compared with the strenuous treadmill exercise regimen usually used [166,208]. These contradictory findings appear to be in line with the notion that skeletal muscle may utilize multiple mitophagy pathways in response to exercise, depending on the exercise type, duration, and/or intensity (Figure 2).

The presence or activation of certain proteins in mitochondria is a crucial connection between damaged mitochondria and the autophagy machinery, and the multiplicity of mitophagy mechanisms may be explained by the possibility that this might happen even in the absence of the mitochondrial membrane potential loss. As previously indicated, BNIP3, which is attached to the mitochondrial membrane, may help trigger the process of mitophagy by binding to LC3 [73]. Elevated BNIP3 expression levels were found in rodent skeletal muscle after intense exercise [209], and similar results were also observed in humans [217]. Following acute exercise, it appears that the creation of the autophagosome via the AMPK–ULK1 axis occurs simultaneously with mitochondrial fission, and fragmented mitochondria were found to be detached from the reticulum and absorbed by autolysosomes [212]. Given the significance of mitochondrial fission in the mitophagy process, a considerable decrease in mitophagy would be anticipated if fission were inhibited. DRP1 heterozygous mice were found to have worse exercise capacity and trainability compared to controls [218]. Although necessary for mitophagy, mitochondrial fission may also serve to shift resources within the mitochondrial reticulum, and fission alone may not always result in mitophagy. Studies have reported that after intense exercise, DRP1 phosphorylation is increased both in skeletal and cardiac muscle cells [208,212]. Moreover, proteomic analyses have shown that AMPK directly phosphorylates MFF [219], while endurance exercise causes MFF phosphorylation [220]. It is conceivable that exercise-induced AMPK activation promotes mitochondrial fission through phosphorylation of MFF and consequent recruitment of DRP1 to support mitophagy, since DRP1 must be recruited from the cytoplasm to the mitochondrial surface to mediate mitochondrial fission [221] and MFF is required for this recruitment (Figure 2).

Finally, a crucial stage in exercise-induced mitophagy is the development of autolysosomes, where the enveloped mitochondria are destroyed. This action seems to be reliant on ULK1 [212]. Mice overexpressing a dominant-negative protein of the AMPK2 subunit displayed reduced production of mitochondria-containing autolysosomes in skeletal muscle in response to acute exercise, which is consistent with the notion that the AMPK–ULK1 axis is important in exercise-induced mitophagy [212]. Additionally, it has been demonstrated that endurance exercise training causes lysosomal biogenesis in skeletal muscle, which might improve the activity of mitophagy [123]. It has been hypothesized that AMPK controls the mitophagy response to exercise by influencing lysosomal biogenesis since transcription factor EB (TFEB), a transcription factor essential for lysosome biogenesis [222], is activated and translocated to the nuclei of skeletal muscle cells as a result of increased contractile activity or exercise [223], and TFEB transcription requires AMPK [224].

## 7. Future Perspectives and Clinical Applications

Considering that mitophagy is the process of selective degradation of damaged or unnecessary mitochondria within the cells, recent studies have suggested that mitochondrial defects may be associated with the pathogenesis of sarcopenia [225] and that mitophagy may play a protective role against the development of sarcopenia [226], attenuating age-related loss of muscle mass and strength [227]. Enhancing mitophagy through various methods, such as the activation of specific signaling pathways or the use of mitochondria-targeted drugs that induce mitophagy [228,229], may be a potential strategy to counteract sarcopenia.

Specifically, a potential strategy for enhancing mitophagy is through the activation of the PINK1/PRKN pathway. As previously described, PINK1 is located in the outer membrane of the healthy mitochondria, and when their integrity is impaired, PINK1 is rapidly degraded. This leads to the activation of PRKN, which then targets the damaged mitochondria for degradation by mitophagosomes. Activating the PINK1/PRKN pathway through genetic manipulation, or with mitochondria-targeted drug delivery systems and utilization of small molecules able to boost mitophagy, may enhance the clearance of damaged mitochondria and improve muscle function in sarcopenia [225,230,231]. Nevertheless, although the potential of enhancing mitophagy as a strategy to counteract sarcopenia is promising, more research is needed to further characterize the mechanisms that modulate mitochondrial homeostasis and other signaling pathways that induce mitophagy in muscle cells. Future studies should also focus on identifying effective, muscle-specific mitochondria-targeted compounds and evaluating their safety, initially in animal models and then in clinical trials.

In addition, mitophagy is thought to be involved in exercise-induced muscle damage and adaptation. During intense exercise, the increased demand for energy leads to increased production of mtROS, which can damage the mitochondria and result in their dysfunction [3,4]. Mitophagy appears to regulate the clearance of these damaged mitochondria and the maintenance of normal mitochondrial function by promoting the formation of new and more functional ones [130,131]. Moreover, mitophagy is activated not only in response to exercise-induced muscle damage but also as an adaptation process to exercise [232]. Indeed, studies in animal models have shown that exercise can increase the levels of the mitophagy-related proteins, PINK1 and PRNK, in muscle tissue [179], while the inhibition of mitophagy can exacerbate muscle damage and impair muscle recovery following exercise. Additionally, it has been suggested that exercise-induced muscle damage may lead to increased production of mitophagy-inducing factors, such as BNIP3, which can trigger the degradation of damaged mitochondria [219,220]. Overall, this evidence supports a compelling need to further understand the association between mitophagy and exercise-induced muscle damage. Hence, future studies should further investigate the mechanisms by which mitophagy is activated in response to exercise and the potential therapeutic effects of enhancing mitophagy, not only on muscle recovery following exercise-induced damage but also on muscle regeneration and remodeling in various myopathies.

## 8. Conclusions

Mitochondria are highly adaptable organelles that may change their structure and function in response to different cellular stressors. Cellular survival depends on these organelles’ natural capacity for adaptation. Indeed, key quality control mechanisms for mitochondria are their de novo synthesis, a dynamic remodeling of their structure, and the eventual elimination of the dysfunctional or damaged mitochondria. Alterations to any of these mechanisms may significantly affect cellular function and even result in disease. Mitophagy, the selective degradation of damaged or redundant mitochondria, has been increasingly studied for its role in muscle maintenance and regeneration. Previous research has shown that mitophagy is important for maintaining the quality and function of mitochondria in muscle cells. Several pathways have been characterized within this context, including the Parkin-mediated mitophagy, activated by damaged or malfunctioning mitochondria and leading to their targeted removal, the BNIP3L/NIX-mediated mitophagy that is involved in the clearance of mitochondria during muscle cell differentiation, and the BNIP3-mediated mitophagy, which has been shown to play a key role in the removal of mitochondria during muscle atrophy. Studies have also shown that mitophagy plays a critical role in muscle regeneration, the repair of muscle tissue, and the restoration of muscle function. The molecular mechanisms of the mitophagy-associated mitochondrial dynamics and network reformation for proper muscle cell regeneration were discussed in this review. Specifically, mitochondrial biogenesis, fission/fusion, and mitophagy, cellular energy/nutrient and stress sensors cooperate to sustain mitochondrial function. Mitochondrial remodeling continually goes through cycles of degeneration and regeneration in response to normal muscle function, exercise, or muscle damage. Muscle regeneration following damage is characterized by a highly regulated, rapid turnover of poor-functioning mitochondria, permitting the synthesis of better-functioning mitochondria to occur. An extensive intracellular mitochondrial modification and mitophagy is required in parallel with satellite cell activation and differentiation to repair or generate new myofibers following muscle damage. However, despite significant progress in the field, there are still gaps in our understanding of the precise mechanisms of mitophagy in muscle cells and its role in muscle maintenance and regeneration. The signaling pathways that regulate mitophagy in muscle cells and the specific molecular players involved are not fully understood. Moreover, the interplay between mitophagy and other cellular processes, such as oxidative stress, inflammation, and autophagy remains to be fully characterized. In addition, different types of muscle damage, such as acute injury or exercise-induced, disease-associated, or aging-related damage may differentially affect mitophagy and its contribution to muscle regeneration. Furthermore, while progress has been made in understanding the role of mitophagy in muscle regeneration using in vitro or animal models, much less is known about this process in humans. Overall, further studies are needed to provide new insights into the regulation of mitophagy in muscle cells and the development of effective strategies for treating muscle injuries and mitochondrial myopathies.
